# Work ability trends 2000–2020 and birth-cohort projections until 2040 in Finland

**DOI:** 10.1177/14034948241228155

**Published:** 2024-02-23

**Authors:** Jouni Lahti, Jaakko Reinikainen, Jukka Kontto, Zhi Zhou, Seppo Koskinen, Mikko Laaksonen, Timo Partonen, Hanna Elonheimo, Annamari Lundqvist, Hanna Tolonen

**Affiliations:** 1 Department of Public Health and Welfare, Finnish Institute for Health and Welfare, Helsinki, Finland; 2 Finnish Centre for Pensions, Helsinki, Finland

**Keywords:** Perceived work ability, work ability score, self-report, projection, trend

## Abstract

*Aims:* To examine age-group and birth-cohort trends in perceived work ability in Finland in 2000–2020 and make projections of perceived work ability up to 2040 based on the observed birth-cohort development. *Methods:* Ten population-representative cross-sectional surveys conducted in Finland between 2000 and 2020 were used (overall *N* = 61,087, range 817–18,956). Self-reported estimates of current work ability in relation to the person’s lifetime best on a scale from zero to ten (0–10) were classified into three groups: limited (0–5), intermediate (6–7), and good (8–10). Multiple imputation was used in projecting work ability. *Results:* Examining past trends by 5-year birth-cohorts born between 1961 and 1995 showed that work ability has declined steadily over time among older birth-cohorts, while in the two younger cohorts a stable development before 2017 and a steep decline between 2017 and 2020 was seen. Trends by 5-year age groups showed a declining trend of good work ability among 20–44-year-olds, a stable trend among 45–54-year-olds, and an improving trend among 55-year-olds and older was observed for the period 2000–2020. Among the under 55-year-olds the prevalence of good work ability ended up around 75% and at 68% among the 55–59-year-olds, 58% among the 60–69-year-olds and 49% among the 70–74-year-olds in 2020. Birth-cohort projections suggested a declining work ability in the future among all age groups included (30–74 years). By 2040, the prevalence of good work ability is projected to decline by 10 to 15 percentage points among 45–74-year-olds. ***Conclusions:*** The projections suggest declining work ability in the future. Efforts to counteract the decline in work ability are needed.

## Introduction

The population in Europe and throughout the developed countries is ageing rapidly. The old-age dependency ratio has been increasing and is projected to continue increasing in the future. For instance, in 2000, there were about four working-aged people for each pension-aged person in Europe, while in 2020 the ratio was about three and in 2050 it is projected to be less than two [1]. This development threatens the financial foundation of welfare states and social protection systems. The strategy in Finland and throughout Europe has focused on raising the age of retirement and increasing employment rates. While longer working careers are of critical importance, in addition, the maintenance of work ability over the course of working life is increasingly important for both individuals and the public economy [2].

Work ability is a multidimensional concept that has traditionally been seen as a balance of health and functioning with the demands of work [3]. In Finland, perceived work ability in the population developed positively in the first decade of the 21^st^ century [4] but this improvement plateaued and partly even reversed from 2011 until 2017 [5]. Differences between age groups are evident, as 90% of the under 50s had good work ability in 2017, compared to less than 60% of people aged 60–69 [5]. At the population level, mental disorders and musculoskeletal diseases are the leading health problems affecting work ability [4]. During the 21^st^ century, sickness absence rates due to mental disorders declined until 2017 in Finland, except among younger women. Since 2017, sickness absence due to mental causes has increased steeply in all age groups [6]. A similar development has been observed in disability pension uptake due to mental causes [7]. At the same time, prevalence of musculoskeletal diseases and other somatic illnesses has declined, but they remain significant causes of work disability, especially among the older age groups [7]. Future projections of work ability are of importance for policy makers to prepare for future challenges and allow time to make the necessary decisions. We aimed to examine age-group and birth-cohort trends in work ability between 2000 and 2020 in Finland and make projections on work ability until 2040 based on the observed development in birth-cohorts.

## Methods

We used data from 10 population-representative cross-sectional health surveys conducted in Finland between 2000 and 2020. The number of participants ranged from 817 to 18,956, the overall number being 61,087 participants. The surveys included the Health 2000 Survey [8], the Regional Health and Wellbeing Study 2010, 2012, 2013, 2014, 2015, 2017 [9], FinHealth 2017 [10] and FinSote 2018 and 2020 [11]. Age was restricted to 20–74, because this age range was available in all surveys. Since there were data from two surveys in 2017, the year number was set to ‘2017.5’ for the Regional Health and Wellbeing Study 2017, which was carried out later in that year than FinHealth 2017 (Table I and Appendix 1).

**Table I.** Number of participants and distributions of work ability by gender and survey year.

	Survey year									
	2000	2010	2012	2013	2014	2015	2017	2017.5	2018	2020
*N*	2775	817	1304	18,956	7390	8069	3552	1589	7478	9157
Age, mean (SD)	30.2	36.0	36.8	37.3	37.9	38.4	41.0	41.7	42.7	44.7
	(5.8)	(8.7)	(9.3)	(9.6)	(9.9)	(10.4)	(9.5)	(9.9)	(10.4)	(10.3)
Gender										
Men, *n* (%)	1362	325	524	7979	3101	3419	1682	683	3175	4016
	(49.1)	(39.8)	(40.2)	(42.1)	(42.0)	(42.4)	(47.4)	(43.0)	(42.5)	(43.9)
Women, *n* (%)	1413	492	780	10,977	4289	4650	1870	906	4303	5141
	(50.9)	(60.2)	(59.8)	(57.9)	(58.0)	(57.6)	(52.6)	(57.0)	(57.5)	(56.1)
Work ability, men										
Good, *n* (%)	1246	272	408	6311	2390	2631	1291	528	2457	3006
	(91.5)	(83.7)	(77.9)	(79.1)	(77.1)	(77.0)	(76.8)	(77.3)	(77.4)	(74.9)
Moderate, *n* (%)	68	25	56	870	357	395	209	80	389	560
	(5.0)	(7.7)	(10.7)	(10.9)	(11.5)	(11.6)	(12.4)	(11.7)	(12.3)	(13.9)
Poor, *n* (%)	33	25	41	526	215	221	97	67	284	395
	(2.4)	(7.7)	(7.8)	(6.6)	(6.9)	(6.5)	(5.8)	(9.8)	(8.9)	(9.8)
Missing, *n* (%)	15	3	19	272	139	172	85	8	45	55
	(1.1)	(0.9)	(3.6)	(3.4)	(4.5)	(5.0)	(5.1)	(1.2)	(1.4)	(1.4)
Work ability, women										
Good, *n* (%)	1298	414	640	8961	3422	3665	1518	721	3426	3852
	(91.9)	(84.1)	(82.1)	(81.6)	(79.8)	(78.8)	(81.2)	(79.6)	(79.6)	(74.9)
Moderate, *n* (%)	82	48	76	1061	440	462	191	109	494	761
	(5.8)	(9.8)	(9.7)	(9.7)	(10.3)	(9.9)	(10.2)	(12.0)	(11.5)	(14.8)
Poor, *n* (%)	23	26	36	610	256	285	102	67	337	471
	(1.6)	(5.3)	(4.6)	(5.6)	(6.0)	(6.1)	(5.5)	(7.4)	(7.8)	(9.2)
Missing, *n* (%)	10	4	28	345	171	238	59	9	46	57
	(0.7)	(0.8)	(3.6)	(3.1)	(4.0)	(5.1)	(3.2)	(1.0)	(1.1)	(1.1)

Work ability was measured similarly in all surveys. Work ability score was derived from a single question asking the respondents to estimate their current work ability in relation to their lifetime best on a scale from zero to ten (0–10), zero indicating completely unable to work and ten work ability at its best. The score was classified into three groups: poor (0–5), moderate (6–7) and good (8–10), following previous procedures. Work ability score has proven to be a valid measure of work ability showing comparable results with a more detailed questionnaire, the work ability index [3].

Other variables derived from surveys used as covariates in the projections were cohabiting: yes or no; educational level: high, intermediate, low; body mass index (BMI): normal weight (BMI <25 kg/m^2^), overweight (BMI ⩾25 kg/m^2^ <30 kg/m^2^) and obese (BMI ⩾30 kg/m^2^); smoking: daily smoker, occasional smoker, non-smoker; and leisure-time physical activity: vigorously active, moderately active, inactive [10]. Limiting longstanding illness (LLI) was measured with two standard questions and dichotomised into those with and without LLI (10). Depression was measured with the Whooley questions [12]: 1. ‘During the past month, have you often been bothered by feeling down, depressed or hopeless?’ 2. ‘During the past month, have you often been bothered by little interest or pleasure in doing things?’ and dichotomised into depressed and not depressed (i.e. no to both questions). Musculoskeletal problems were measured with somewhat varying questions in the different surveys; however, they allowed formation of a dichotomised variable separating those with musculoskeletal disease diagnosed by a doctor during the past year and those without a musculoskeletal disease. Not all these covariates were available from all surveys, therefore missing variables are presented in Appendix 1.

## Statistical methods

We examined trends in work ability among women and men between 2000 and 2020 by 5-year birth-cohorts and by 5-year age groups within the age range from 20 to 74 years. To illustrate the expected future development of different birth-cohorts, we examined trends by 5-year birth-cohorts born between 1961 and 1995, which provided sufficient data points to enable reliable projections estimating the future work ability in 5-year age groups. Only the years when a birth-cohort was within the age range of 20–74 were taken into account to obtain the projected prevalence of work ability within age range 45–74 years in 2040, 40–74 years in 2035, 35–74 in 2030 and 30–74 in 2025. For 20–29-year-olds no projections are included since there were insufficient data points to indicate past trends for this birth-cohort. Overall, work ability trends between 2000 and 2020 were similar between the genders (Appendices 2 and 3), thus we pooled women and men for the main analyses. As the COVID-19 pandemic may have had a strong period effect on work ability, we made the projections with and without data from the year 2020. Projections for work ability were carried out using a multiple imputation method [13]. We created data frames for the future surveys, so that the age and gender distributions were matched with the national population forecasts in the corresponding years (https://stat.fi/en/statistics/vaenn) and the other variables were missing data. This allowed us to take into account the expected changes in the population structure and possible temporal changes in the levels of covariates, thereby increasing the precision of the projections based on 5-year birth-cohort development in work ability. Logistic- and polytomous logistic regression models were used to impute binary and categorical variables, respectively, and the models were selected according to the Bayesian information criterion. Each of the aforementioned variables having missing data was considered as the outcome variable in turn, and other variables, including the survey year, were used as covariates in the imputation models. The missing future data, as well as missing values in the past surveys, were imputed multiple times at the individual-level and then pooled to obtain the population-level prevalence estimates for each year. Sampling weights were used in the calculation of prevalence from the past surveys to take into account the sampling designs and to adjust for non-participation. The R package mice [14] was used for multiple imputation with 50 imputed data sets and 10 iterations.

## Results

Descriptive information on numbers and mean age of participants and the distribution of work ability by gender and survey year are presented in Table I. In addition, the distribution of covariates is presented in Appendix 1.

### Trends of work ability by birth-cohort and projections until 2040

**Figure 1.**
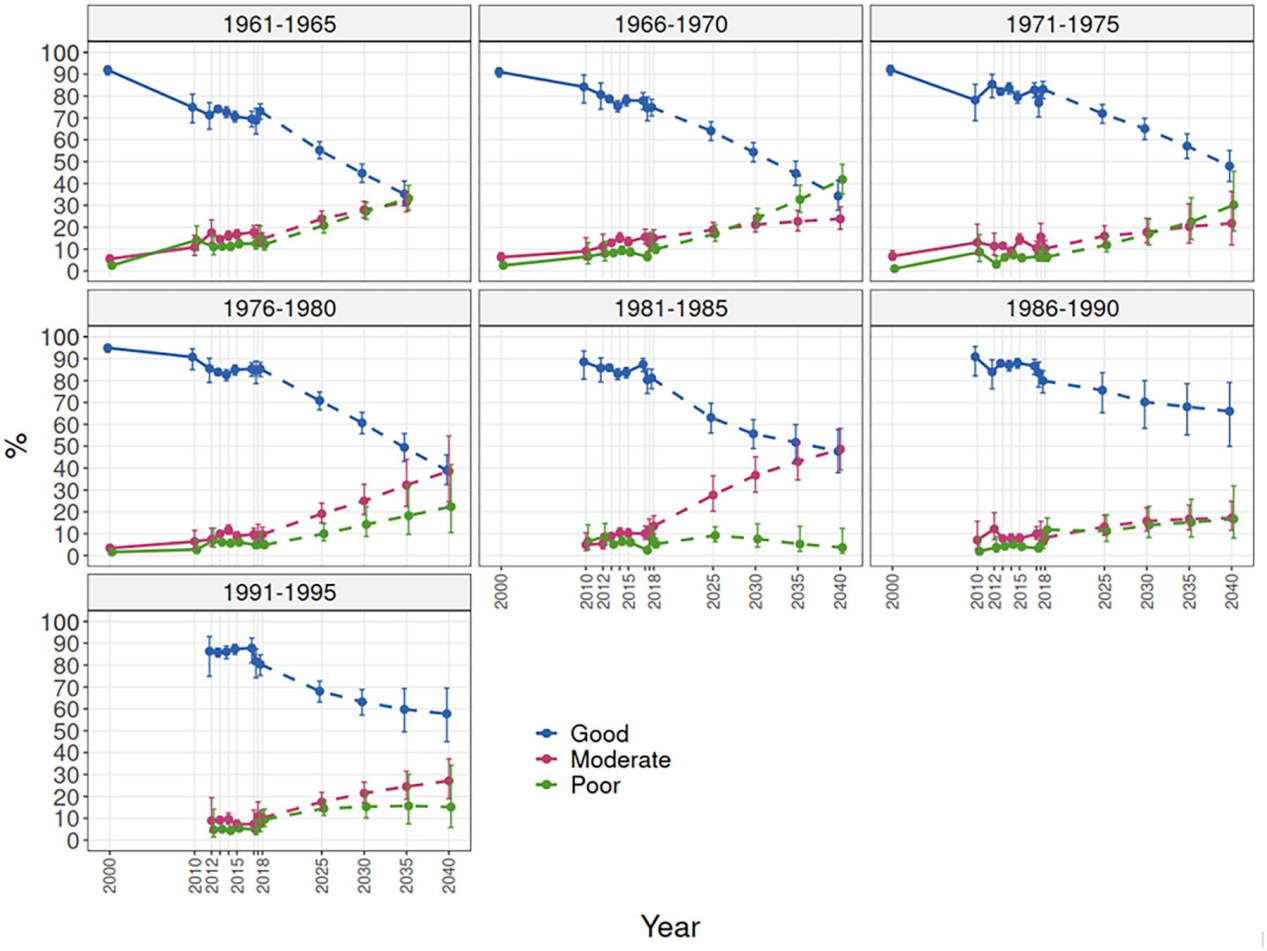
Work ability trends by birth-cohort 2000–2018 and projections until 2040.

**Figure 2.**
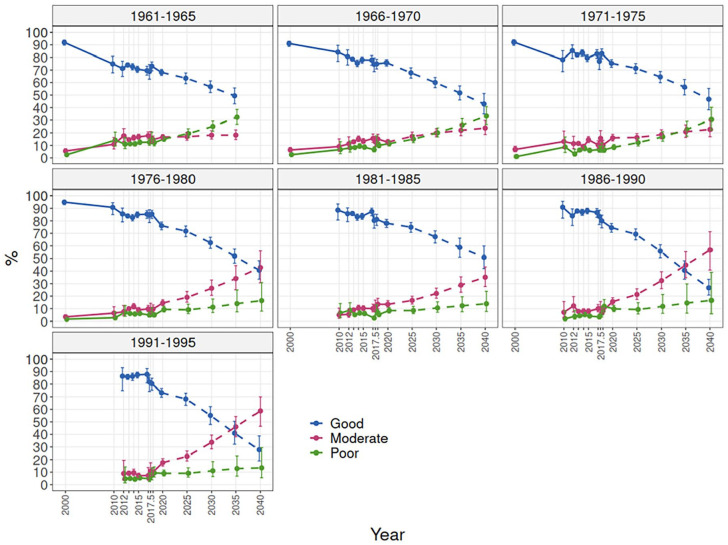
Work ability trends by birth-cohort 2000–2020 and projections until 2040.

Figure 1 presents past trends 2000–2018 (solid lines) and projections 2025–2040 (dashed lines). Examining work ability trends by 5-year birth-cohorts showed that the decline in good work ability was similar and quite linear during the period 2000–2020 in the older cohorts, and in the two younger cohorts the development was quite stable from 2010 to 2017, after which the prevalence of good work ability declined steeply (Figures 1 and 2). Consequently, the prevalence of poor and moderate work ability increased in all cohorts. Among the younger cohorts, the increase was more evident in the share of moderate work ability, whereas among the older cohorts the share of poor work ability increased more. Projections based on the observed development in birth-cohorts during the period 2000–2018 (Figure 1) show a declining development in all birth-cohorts. Those cohorts born 1961–1975 have similar projected development, whereas those born in 1976–1985 have a somewhat lower projected prevalence of good work ability at the same age.

A scenario including the COVID-19 impacted year, 2020, showed 30% estimates for good work ability in 2040 among the younger cohorts born in 1986–1995 (Figure 2). The projections for birth-cohorts born in 1971–1985 showed similar patterns when including or excluding the year 2020, and a somewhat slower decline for older cohorts born in 1961–1970 when including 2020. Among the younger cohorts, the projections showed a greater increase in moderate work ability than in poor work ability, whereas among older cohorts a stronger increase in poor work ability was seen.

## Trends of work ability by 5-year age groups and projections until 2040

**Figure 3.**
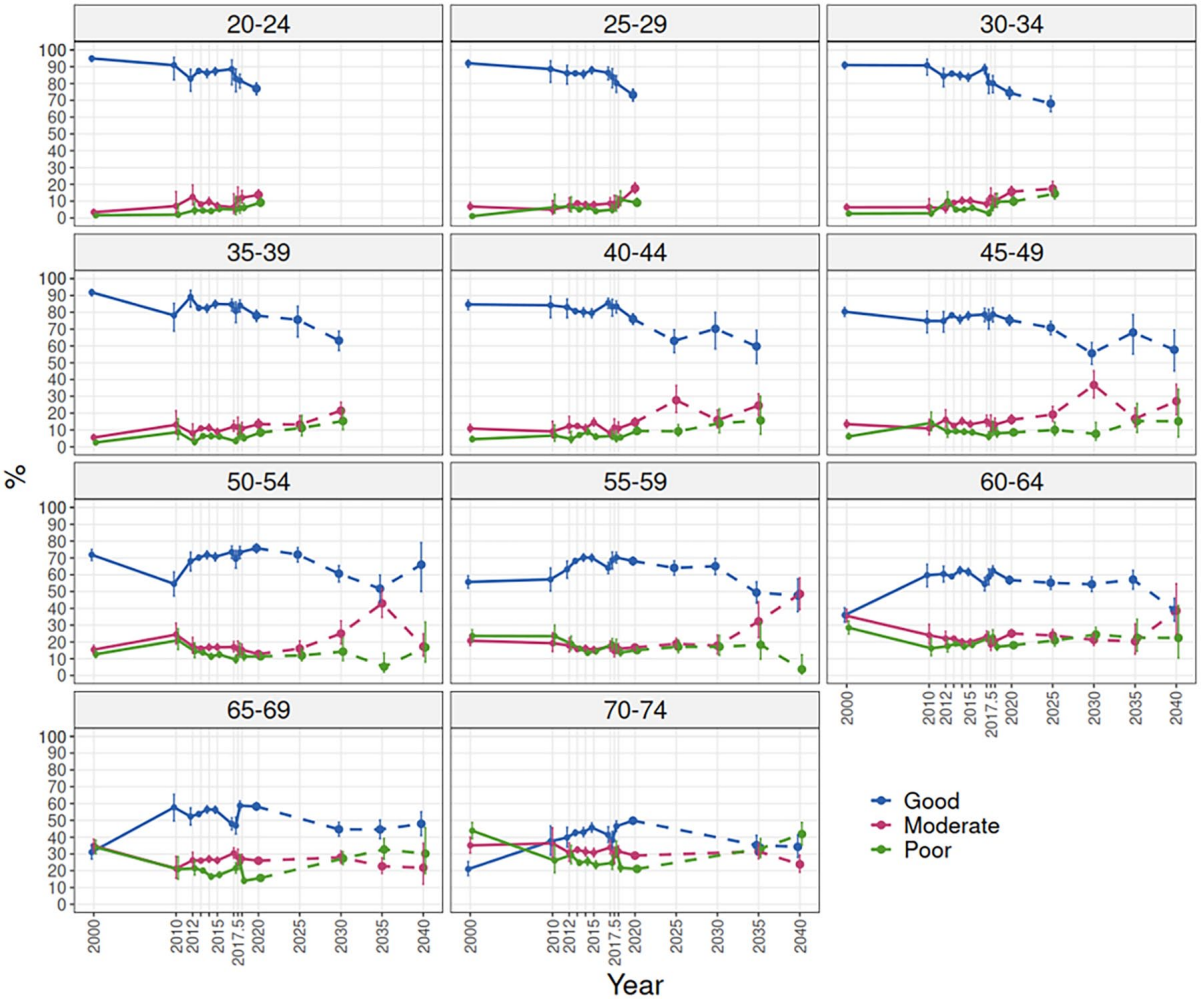
Work ability trends by 5-year age groups 2000–2020 and projections 2025–2040 based on birth-cohorts.

Looking at work ability by 5-year age groups (Figure 3) showed that there was a downward trend among 20–29-year-olds from 2000, but the decline first plateaued around 2013 and then continued steeply from 2017 onwards, the prevalence of good work ability ending up at around 75% in 2020. Among 30–44-year-olds, the development was declining slightly and fairly similar, the prevalence of good work ability ending up at around 75% in 2020. Among 45–54-year-olds, work ability declined from 2000 to 2010, then increased until 2020, the prevalence of good work ability ending up at the same level as in 2000, that is, around 75%. Among the 55-year-olds and older, work ability improved, the prevalence of good work ability ending up around 70% among the 55–59-year-olds, 60% among the 60–69-year-olds and 50% among the 70–74-year-olds in 2020. The prevalence rates of good work ability by age across all the survey years showed that the decline was relatively slow between ages 20 and 45 and steeper thereafter (Appendix 4).

Including the birth-cohort projections (Figure 1) in the age-group trend figures 2000–2020 illustrated that the prevalence of good work ability is projected to decline during the period 2020–2040 in all age groups included in the projections (Figure 3). Among 30–44-year-olds, the prevalence of good work ability is projected to decline by approximately five percentage points in 5 years. Among the older cohorts the projections show greater fluctuation over time and the projected decline is less steep among 45–59-year-olds and even more so among the 60–74-year-olds. By 2040, the prevalence of good work ability is projected to decline by 10 to 15 percentage points among 45–74-year-olds.

## Discussion

The main findings of this study can be summarised as follows. Examining past trends of birth-cohorts born between 1961 and 1985 showed that perceived work ability declined steadily among older birth-cohorts during the period 2000–2020, while the younger cohorts born in 1986–1995 had a stable development before 2017 and a steep decline between 2017 and 2020. Age-group trends during the period 2000–2020 showed a declining work ability among younger age groups, a stable work ability among the middle-aged, and an improving work ability among the older age groups. In 2020, among the younger and the middle-aged, three out of four perceived their work ability as good. The share of good work ability declined towards older age groups, half having good work ability among the 70–74-year-olds. According to the birth-cohort projections, the stable development in work ability among the middle-aged, and the improving work ability among older groups during the two first decades of the 21^st^ century are likely to reverse in the near future.

Explanations for these trends can be sought in the structural changes of education and labour markets. Technological development has been increasingly rapid, as has the pace of educational upgrading and globalisation, leading to new organisational structures characterised by higher job insecurity and greater complexity of work, which are likely to increase work-related stress. For instance, work strain has increased from 1995 to 2015 in many European countries mainly due to increasing psychological demands, however, less so in countries such as Finland who have invested more money in active labour-market policy, for example, in training and job-search assistance for the unemployed [15]. Younger cohorts may have different expectations and attitudes towards work than previous cohorts [16], which may negatively affect perceived work ability. The decreased perceived work ability seen the among younger cohorts has coincided with the declining results and widening socioeconomic gap in the academic performance of 15-year-old Finnish children [17], suggesting that the capabilities acquired in vocational education and training may not be fully meeting the job requirements of today’s society. Education policy should also invest in supporting the psychosocial development of students in schools as well as the successful completion of education and transition into the labour market [18].

The present findings among younger cohorts are in line with the increased sickness absence and disability pensions due to mental health problems found elsewhere [6,7]. Mental health problems before working age may have a long-term influence in health and work later in life [19]. One study showed that among adolescents, self-reported depressive symptoms increased in Finland between 2000 and 2011 [20], whereas another study showed no increase between 1998 and 2008 using a different self-reported measure [21]. However, self-reported symptoms are not equal to clinically verified disorders where an assessment of functioning is needed for diagnosis, and symptoms must affect work, social or domestic activities, and necessitate clinical attention. Treatment of mental disorders such as anxiety disorders among adolescents [22] as well as antidepressant medication [23] has increased, but this trend may reflect changing treatment practices [24] and better identification of mental disorders. A review on secular trends found that mental health problems among adolescents and children increased in high-income countries during the first decade of the 21^st^ century [25]. In addition, a large European study examining trends in general disability during the period 2002–2016 found increased levels of disability among younger cohorts born 1970–2000 compared to previous cohorts [26]. Although mental health disorders are better recognised, sufficient resources have not been allocated to appropriate treatment such as psychotherapy and multi-professional rehabilitation to meet the growing need.

Based on the projections by birth-cohorts, the stable development in work ability among the middle-aged, and the improving work ability among the older Finnish population seen between 2000 and 2020 are likely to plateau and even reverse in the near future. In addition, the decline in work ability among 30–44-year-olds is projected to continue. In 2040, for instance, among persons aged 55 years or over, half or less are projected to have good work ability, which is about 10 to 15 percentage-points lower than today. This projected development is due to younger cohorts perceiving their work ability to be poorer than the previous cohorts when entering working life and the fact that work ability will decrease in each cohort as health problems increase and functioning declines with age [4]. Future development of mental disorders and musculoskeletal problems, but also their countermeasures and health promotion, will largely determine the changes in work ability of the population. For instance, promoting healthy lifestyles for improving the physical activity and fitness of the population may prove useful as they have shown importance for work ability [27]. There is much potential as only a third of the adult Finnish population fulfils the current recommendation for health-enhancing physical activity and, furthermore, cardiorespiratory fitness has been declining for the past 40 years in many countries such as Finland [28], most likely having negative consequences on work ability.

Beyond factors related to health, education, working conditions and the labour market, societal factors, such as changes in the overall economic development and changes in legislation, may also play a role in the health and work ability of the work force [29]. Sufficient resources should be allocated to a wide range of preventive measures, including education policy, to improve learning outcomes, policies improving job security, active labour-market programmes, support for youth employment, job redesign programmes and better access to mental health care. Many new practices have been developed in the Work ability programme (Ministry of Social Affairs and Health (stm.fi)) aimed at increasing the employment rates of those with reduced work ability by integrating multidisciplinary work ability support into primary social and health care services. In addition, The National Mental Health Strategy 2020–2030 (https://stm.fi/en/mental-health-policy-guidelines) has a broad multidisciplinary approach including a focus on improving mental health among children and young people. The strategy also includes the mental health at work programme (https://hyvatyo.ttl.fi/en/mindandwork), with several toolkits for workplaces. Thus, important steps have already been taken in Finland to tackle the observed negative developments. Nevertheless, the underlying causes behind these developments should be examined in more detail and different future scenarios should be examined with diverse development scenarios of factors affecting work ability. Since in the present study we did not assume any scenarios for the included covariates that would differ from their observed trends, their role was mainly to increase the precision of the work ability projections. A topic for future research will be to consider the impact of different scenarios of key factors affecting work ability.

### Methodological considerations

We analysed a widely used and valid measure of work ability [3]. In the multiple imputation analyses we were able to take into account the effects of the selected health, lifestyle and sociodemographic covariates, and their expected development over time as well as the expected changes in population structure producing more reliable estimates. However, projections are prone to uncertainty, and the observed development of work ability by birth-cohorts, especially for the younger cohorts with only a short follow-up period (2012–2020) was strongly affected by a single or a few measurement points, leading to ambiguous projections. The projections for birth-cohorts born 1971–1985 showed similar patterns when including or excluding the year 2020, and a somewhat slower decline for older cohorts born 1961–1970 when including 2020. In contrast, for the two younger cohorts born 1986–1995, projections showed a very steep decline, with the proportion of good work ability ending up as low as 30% in 2040 when including data from 2020, whereas when excluding data from 2020 a slower decline was projected, and the prevalence of good work ability in 2040 was around 60% to 65%, which is a more plausible scenario. The COVID-19 epidemic seems to have had a disproportionate negative effect on the perceived work ability of younger cohorts. Previous evidence suggests, for instance, that mental health problems such as anxiety related to COVID-19 increased more among the younger population [30].

## Conclusions

The observed decline in perceived work ability among the younger age groups and the projected decline in the prevalence of good work ability among the middle-aged and older cohorts are concerning. Efforts to counteract the projected decline are urgently needed. Further studies should examine the underlying causes behind these developments. Furthermore, future scenarios considering different development patterns of major factors affecting work ability are needed.

## Supplemental Material

sj-docx-1-sjp-10.1177_14034948241228155 – Supplemental material for Work ability trends 2000–2020 and birth-cohort projections until 2040 in Finland Supplemental material, sj-docx-1-sjp-10.1177_14034948241228155 for Work ability trends 2000–2020 and birth-cohort projections until 2040 in Finland by Jouni Lahti, Jaakko Reinikainen, Jukka Kontto, Zhi Zhou, Seppo Koskinen, Mikko Laaksonen, Timo Partonen, Hanna Elonheimo, Annamari Lundqvist and Hanna Tolonen in Scandinavian Journal of Public Health

## References

[1] European Commission, Eurostat, Corselli-Nordblad L , et al. Ageing Europe – looking at the lives of older people in the EU: 2020 edition. Luxembourg: Publications Office of the European Union, 2020.

[2] Virtanen M. Towards sustainable work and longer working lives. Scand J Public Health 2018;46:287–9. 10.1177/1403494818765394 29732963

[3] El Fassi M Bocquet V Majery N , et al. Work ability assessment in a worker population: comparison and determinants of Work Ability Index and Work Ability score. BMC Public Health 2013;13:305. 23565883 10.1186/1471-2458-13-305 PMC3637198

[4] Gould R Koskinen S Sainio P , et al. Työkyky. In: Koskinen S Lundqvist A Ristiluoma N (eds) Health, functional capacity and welfare on Finland in 2011. THL Report 68/2012, Helsinki, 2012.

[5] Koskinen S Sainio P. Work ability in Finland: earlier positive time trend has reversed. Eur J Public Health 2018;28:cky213.741.

[6] Blomgren J Perhoniemi R. Increase in sickness absence due to mental disorders in Finland: trends by gender, age and diagnostic group in 2005–2019. Scand J Public Health 2022;50:318–22. 10.1177/1403494821993705 PMC9096587 33615899

[7] Laaksonen M Blomgren J Perhoniemi R. Mielenterveyssyistä alkavat eläkkeet ovat yleistyneet nuorilla mutta vähentyneet vanhemmissa ikäryhmissä [Pensions starting for mental health reasons have become more common among young people, but have decreased among older age groups]. Suom Lääkäril 2021;76:1889–97.

[8] Heistaro S (ed). Methodology report – *Health 2000 Survey*. Publication of the National Public Health Institute B26/2008. Helsinki: National Public Health Institute (KTL), 2008.

[9] Härkänen T Kaikkonen R Virtala E , et al. Inverse probability weighting and doubly robust methods in correcting the effects of non-response in the reimbursed medication and self-reported turnout estimates in the ATH survey. BMC Public Health 2014;14:1150. 25373328 10.1186/1471-2458-14-1150 PMC4246429

[10] Borodulin K Sääksjärvi K (eds). FinHealth 2017 Study – methods. Report 17/2019 ed. Helsinki, Finland: Finnish Institute for Health and Welfare, 2019.

[11] Parikka S Koskela T Ikonen J , et al. The adult population’s well-being, health and services – FinSote 2020 regional differences in the service experiences and well-being of adults. Statistical report 16/2021. Finnish Institute for Health and Welfare: Helsinki, Finland, 2021.

[12] Whooley MA Avins AL Miranda J , et al. Case-finding instruments for depression: two questions are as good as many. J Gen Intern Med 1997;12:439–45. 10.1046/j.1525-1497.1997.00076.x PMC1497134 9229283

[13] Reinikainen J Härkänen T Tolonen H. Projections for obesity, smoking and hypertension based on multiple imputation. Scand J Public Health 2023;51:829–34. 10.1177/14034948211061014 PMC10350717 34904475

[14] Van Buuren S Groothuis-Oudshoorn K . Mice: multivariate imputation by chained equations in R. J Stat Software 2011;45:1–67.

[15] Rigó M Dragano N Wahrendorf M , et al. Long-term trends in psychosocial working conditions in Europe – the role of labor market policies. Eur J Public Health 2022;32:384–91. 10.1093/eurpub/ckac038 PMC9159322 35472073

[16] Weeks KP Schaffert C. Generational differences in definitions of meaningful work: a mixed methods study. J Bus Ethics 2019;156:1045–61.

[17] Muench R Wieczorek O Dressler J. Equity lost: Sweden and Finland in the struggle for PISA scores. Eur Educ Res J 2022;22:413–32.

[18] Veldman K Reijneveld SA Ortiz JA , et al. Mental health trajectories from childhood to young adulthood affect the educational and employment status of young adults: results from the TRAILS study. J Epidemiol Community Health 2015;69:588–93. 10.1136/jech-2014-204421 25667302

[19] Wickrama KA Wickrama T Lott R. Heterogeneity in youth depressive symptom trajectories: social stratification and implications for young adult physical health. J Adolesc Health 2009;45:335–43. 10.1016/j.jadohealth.2009.04.018 19766937

[20] Torikka A Kaltiala-Heino R Rimpelä A , et al. Self-reported depression is increasing among socio-economically disadvantaged adolescents – repeated cross-sectional surveys from Finland from 2000 to 2011. BMC Public Health 2014;14:408. 24775269 10.1186/1471-2458-14-408 PMC4031153

[21] Sourander A Koskelainen M Niemelä S , et al. Changes in adolescents’ mental health and use of alcohol and tobacco: a 10-year time-trend study of Finnish adolescents. Eur Child Adolesc Psychiatry. 2012;21:665–71. 10.1007/s00787-012-0303-8 22782292

[22] Khanal P Ståhlberg T Luntamo T , et al. Time trends in treated incidence, sociodemographic risk factors and comorbidities: a Finnish nationwide study on anxiety disorders. BMC Psychiatry 2022;22:144. 35193518 10.1186/s12888-022-03743-3 PMC8864838

[23] SLTFSM. Suomen lääketilasto 2019: Finnish statistics of medicines 2019. Helsinki: Fimea ja Kela, 2020.

[24] Dowrick C Frances A. Medicalising unhappiness: new classification of depression risks more patients being put on drug treatment from which they will not benefit. BMJ 2013;347:f7140. 10.1136/bmj.f7140 24322400

[25] Collishaw S. Annual research review: secular trends in child and adolescent mental health. J Child Psychol Psychiatry 2015;56:370–93. 10.1111/jcpp.12372 25496340

[26] Beller J Epping J. Disability trends in Europe by age-period-cohort analysis: increasing disability in younger cohorts. Disabil Health J 2021;14:100948. 32690322 10.1016/j.dhjo.2020.100948

[27] Kerner I Rakovac M Lazinica B. Leisure-time physical activity and absenteeism. Arh Hig Rada Toksikol 2017;68:159–70. 10.1515/aiht-2017-68-2963 28976887

[28] Lamoureux NR Fitzgerald JS Norton KI , et al. Temporal trends in the cardiorespiratory fitness of 2,525,827 adults between 1967 and 2016: a systematic review. Sports Med 2019;49:41–55. 30390202 10.1007/s40279-018-1017-y

[29] Kaltenbrunner Bernitz B Grees N Jakobsson Randers M , et al. Young adults on disability benefits in 7 countries. Scand J Public Health 2013;41:3–26. 10.1177/1403494813496931 24077622

[30] Jia R Ayling K Chalder T , et al. The prevalence, incidence, prognosis and risk factors for symptoms of depression and anxiety in a UK cohort during the COVID-19 pandemic. BJPsych Open 2022;8:e64. 10.1192/bjo.2022.34 PMC8914134 35256024

