# Supplementary material for: Work ability trends 2000–2020 and birth-cohort projections until 2040 in Finland
Source: Scand J Public Health. 2024 Feb 23;53(1):62–70. doi: 10.1177/14034948241228155 (PMC11742703; doi:10.1177/14034948241228155)
Supplement: sj-docx-1-sjp-10.1177_14034948241228155 – Supplemental material for Work ability trends 2000–2020 and birth-cohort projections until 2040 in Finland [file sj-docx-1-sjp-10.1177_14034948241228155.docx]

## Supplementary table 1. Distributions of background variables.

|  | **Survey year** | | | | | | | | | |
| --- | --- | --- | --- | --- | --- | --- | --- | --- | --- | --- |
|  | **2000^a^** | **2010^b^** | **2012^b^** | **2013^b^** | **2014^b^** | **2015^b^** | **2017^c^** | **2017.5^b^** | **2018^d^** | **2020^d^** |
| **N** | 2775 | 817 | 1304 | 18956 | 7390 | 8069 | 3552 | 1589 | 7478 | 9157 |
| **Education level** |  |  |  |  |  |  |  |  |  |  |
| Low, n (%) | 1126 (40.6) | 290  (35.5) | 494  (37.9) | 7409 (39.1) | 2910 (39.4) | 3088 (38.3) | 1154 (32.5) | 636  (40.0) | 2708 (36.2) | 3291 (35.9) |
| Intermediate, n (%) | 891  (32.1) | 296  (36.2) | 388  (29.8) | 6006 (31.7) | 2333 (31.6) | 2575 (31.9) | 1106 (31.1) | 478  (30.1) | 2436 (32.6) | 3259 (35.6) |
| High, n (%) | 738  (26.6) | 223  (27.3) | 404  (31.0) | 5357 (28.3) | 2084 (28.2) | 2262 (28.0) | 1142 (32.2) | 462  (29.1) | 2254 (30.1) | 2526 (27.6) |
| Missing, n (%) | 20  (0.7) | 8  (1.0) | 18  (1.4) | 184  (1.0) | 63  (0.9) | 144  (1.8) | 150  (4.2) | 13  (0.8) | 80  (1.1) | 81  (0.9) |
| **Living alone** |  |  |  |  |  |  |  |  |  |  |
| No, n (%) | 2159 (77.8) | 642  (78.6) | 1065 (81.7) | 15557 (82.1) | 5993 (81.1) | 6491 (80.4) | 2854 (80.3) | 1255 (79.0) | 5977 (79.9) | 6917 (75.5) |
| Yes, n (%) | 605  (21.8) | 152  (18.6) | 209  (16.0) | 2958 (15.6) | 1197 (16.2) | 1329 (16.5) | 666  (18.8) | 311  (19.6) | 1461  (19.5) | 1771 (19.3) |
| Missing, n (%) | 11  (0.4) | 23  (2.8) | 30  (2.3) | 441  (2.3) | 200  (2.7) | 249  (3.1) | 32  (0.9) | 23  (1.4) | 40  (0.5) | 469  (5.1) |
| **Depression** |  |  |  |  |  |  |  |  |  |  |
| No, n (%) |  | 599  (73.3) | 905  (69.4) | 13336 (70.4) | 5160 (69.8) | 5580 (69.2) | 2518 (70.9) | 1152 (72.5) |  |  |
| Yes, n (%) |  | 212  (25.9) | 361  (27.7) | 5129 (27.1) | 1972 (26.7) | 2123 (26.3) | 897  (25.3) | 425  (26.7) |  |  |
| Missing, n (%) |  | 6  (0.7) | 38  (2.9) | 491  (2.6) | 258  (3.5) | 366  (4.5) | 137  (3.9) | 12  (0.8) |  |  |
| **Musculoskeletal diseases** |  |  |  |  |  |  |  |  |  |  |
| No, n (%) | 2501 (90.1) | 661  (80.9) | 1045 (80.1) | 14933 (78.8) | 5799 (78.5) | 6257 (77.5) | 2783 (78.4) | 1368 (86.1) | 6364 (85.1) |  |
| Yes, n (%) | 255  (9.2) | 139  (17.0) | 223  (17.1) | 3510 (18.5) | 1394 (18.9) | 1558 (19.3) | 588  (16.6) | 221  (13.9) | 1114 (14.9) |  |
| Missing, n (%) | 19  (0.7) | 17  (2.1) | 36  (2.8) | 513  (2.7) | 197  (2.7) | 254  (3.1) | 181  (5.1) | 0  (0.0) | 0  (0.0) |  |
| **Limiting long standing illness** |  |  |  |  |  |  |  |  |  |  |
| No, n (%) | 1996 (71.9) |  |  |  |  |  | 2658 (74.8) | 1197  (75.3) | 5374  (71.9) | 6386  (69.7) |
| Yes, n (%) | 771  (27.8) |  |  |  |  |  | 804  (22.6) | 364  (22.9) | 1921 (25.7) | 2534 (27.7) |
| Missing, n (%) | 8  (0.3) |  |  |  |  |  | 90  (2.5) | 28  (1.8) | 183  (2.4) | 237  (2.6) |
| **Body mass index** |  |  |  |  |  |  |  |  |  |  |
| Normal weight, n (%) | 1635 (58.9) | 432  (52.9) | 666  (51.1) | 9343 (49.3) | 3655 (49.5) | 3869 (47.9) | 1418 (39.9) | 703  (44.2) | 3072 (41.1) | 3380 (36.9) |
| Overweight, n (%) | 819  (29.5) | 249  (30.5) | 390  (29.9) | 6158 (32.5) | 2345 (31.7) | 2608 (32.3) | 1287 (36.2) | 568  (35.7) | 2691 (36.0) | 3350 (36.6) |
| Obese, n (%) | 293  (10.6) | 115  (14.1) | 202  (15.5) | 2912 (15.4) | 1156 (15.6) | 1309 (16.2) | 833  (23.5) | 296  (18.6) | 1548 (20.7) | 2238  (24.4) |
| Missing, n (%) | 28  (1.0) | 21  (2.6) | 46  (3.5) | 543  (2.9) | 234  (3.2) | 283  (3.5) | 14  (0.4) | 22  (1.4) | 167  (2.2) | 189  (2.1) |
| **Physical activity** |  |  |  |  |  |  |  |  |  |  |
| Inactive | 677  (24.4) | 226  (27.7) | 305  (23.4) | 4434 (23.4) | 1606 (21.7) | 1937 (24.0) | 810  (22.8) | 333  (21.0) | 1397 (18.7) |  |
| Moderately active | 1179  (42.5) | 384  (47.0) | 574  (44.0) | 8516 (44.9) | 3489 (47.2) | 3455 (42.8) | 1443 (40.6) | 813  (51.2) | 3808 (50.9) |  |
| Vigorously active | 640  (23.1) | 194  (23.7) | 392  (30.1) | 5570 (29.4) | 2075 (28.1) | 2413 (29.9) | 1242 (35.0) | 427  (26.9) | 1959 (26.2) |  |
| Missing, n (%) | 279  (10.1) | 13  (1.6) | 33  (2.5) | 436  (2.3) | 220  (3.0) | 264  (3.3) | 57  (1.6) | 16  (1.0) | 314  (4.2) |  |
| **Smoking status** |  |  |  |  |  |  |  |  |  |  |
| Daily smoker, n (%) | 774  (27.9) | 130  (15.9) | 215  (16.5) | 2833 (14.9) | 1093 (14.8) | 1116 (13.8) | 492  (13.9) | 191  (12.0) | 932  (12.5) | 1010 (11.0) |
| Occasional smoker, n (%) | 280  (10.1) | 71  (8.7) | 126  (9.7) | 2049 (10.8) | 771  (10.4) | 840  (10.4) | 342  (9.6) | 155  (9.8) | 554  (7.4) | 645  (7.0) |
| Non-smoker, n (%) | 1712 (61.7) | 610  (74.7) | 932  (71.5) | 13651 (72.0) | 5296 (71.7) | 5848 (72.5) | 2700 (76.0) | 1223 (77.0) | 5610 (75.0) | 7168 (78.3) |
| Missing, n (%) | 9  (0.3) | 6  (0.7) | 31  (2.4) | 423  (2.2) | 230  (3.1) | 265  (3.3) | 18  (0.5) | 20  (1.3) | 382  (5.1) | 334  (3.6) |

^a^ Health 2000

^b^ the Regional Health and Wellbeing Study

^c^ FinHealth 2017

^d^ FinSote


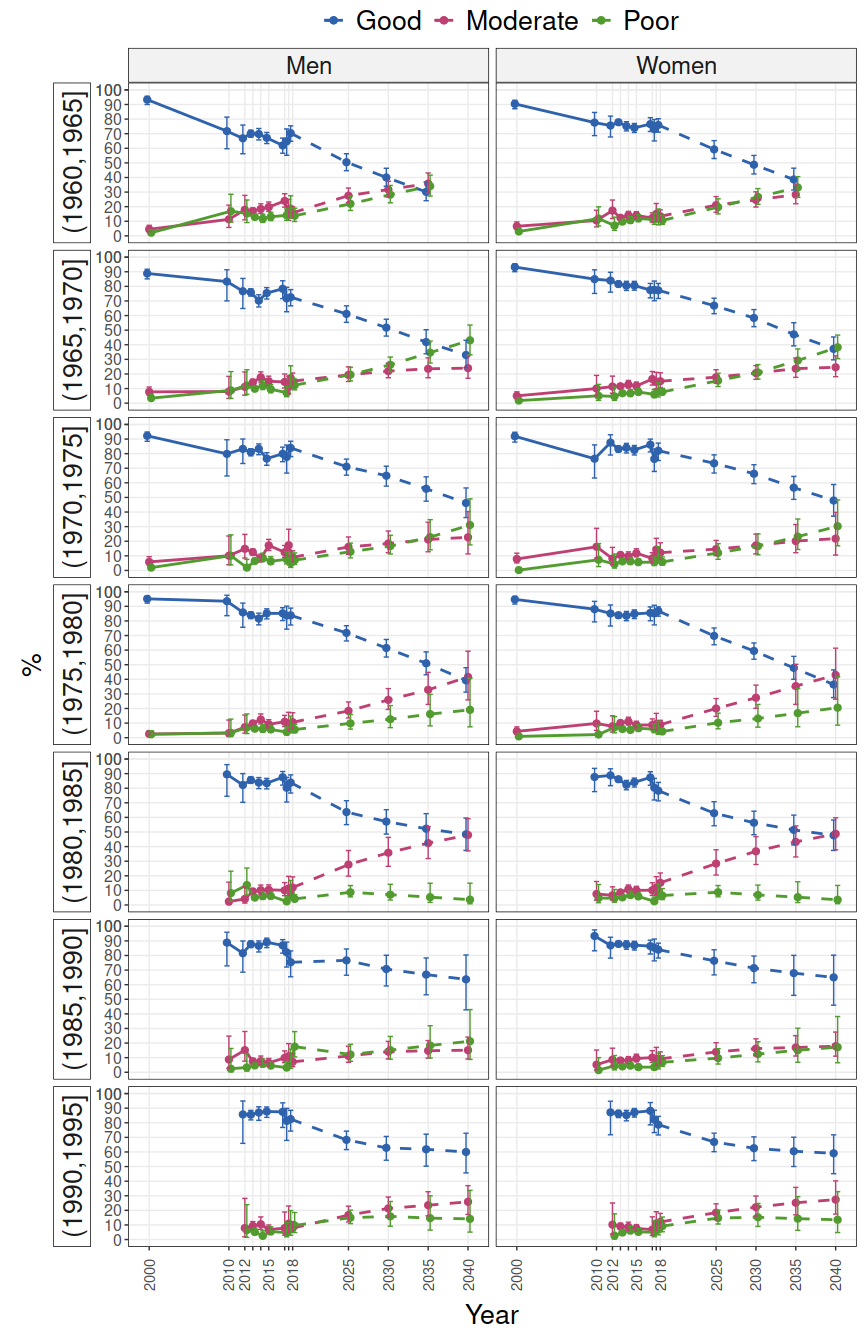


Supplementary figure 2. Work ability trends by birth-cohorts 2000-2018 and projections until 2040 by gender.


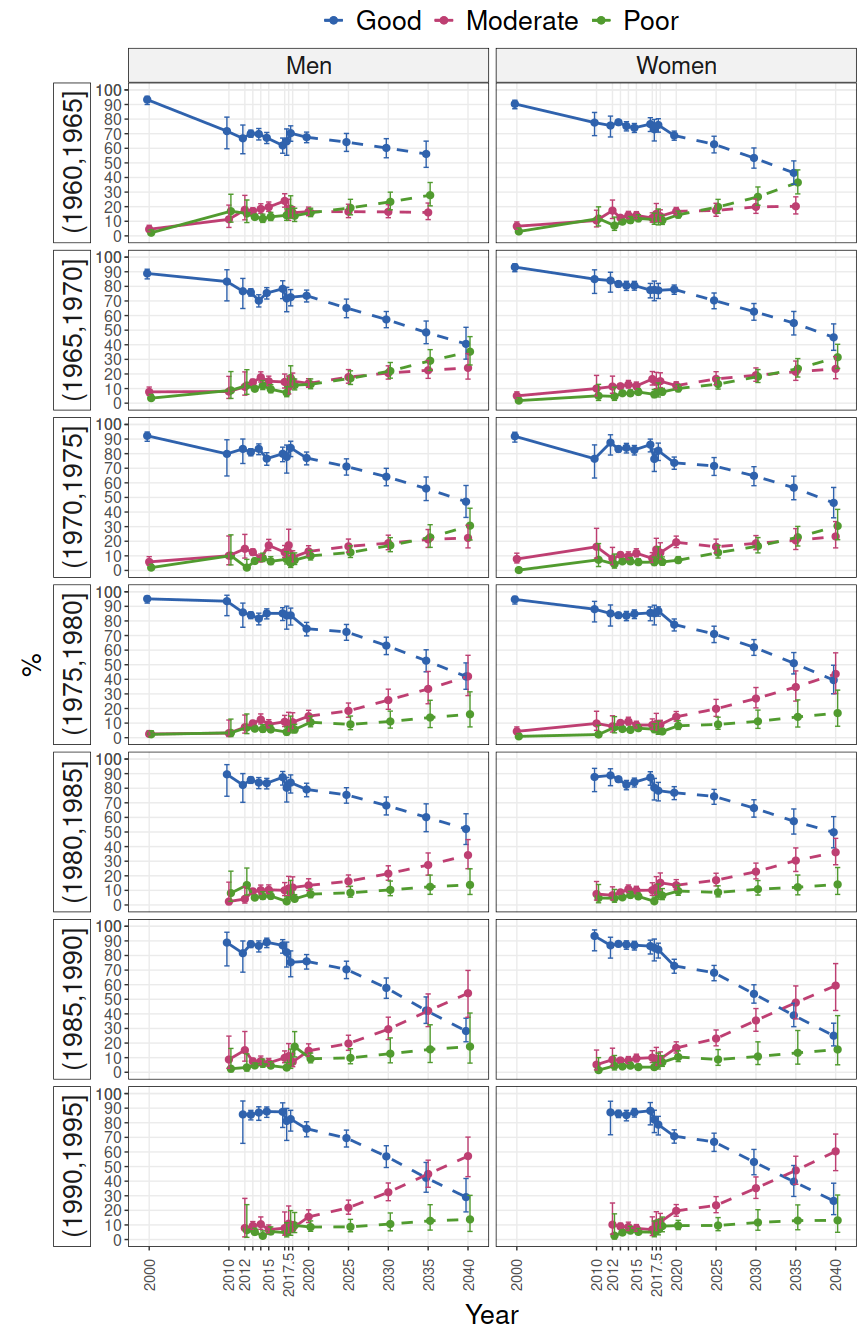
Supplementary figure 3. Work ability trends by birth-cohorts 2000-2020 and projections until 2040 by gender.


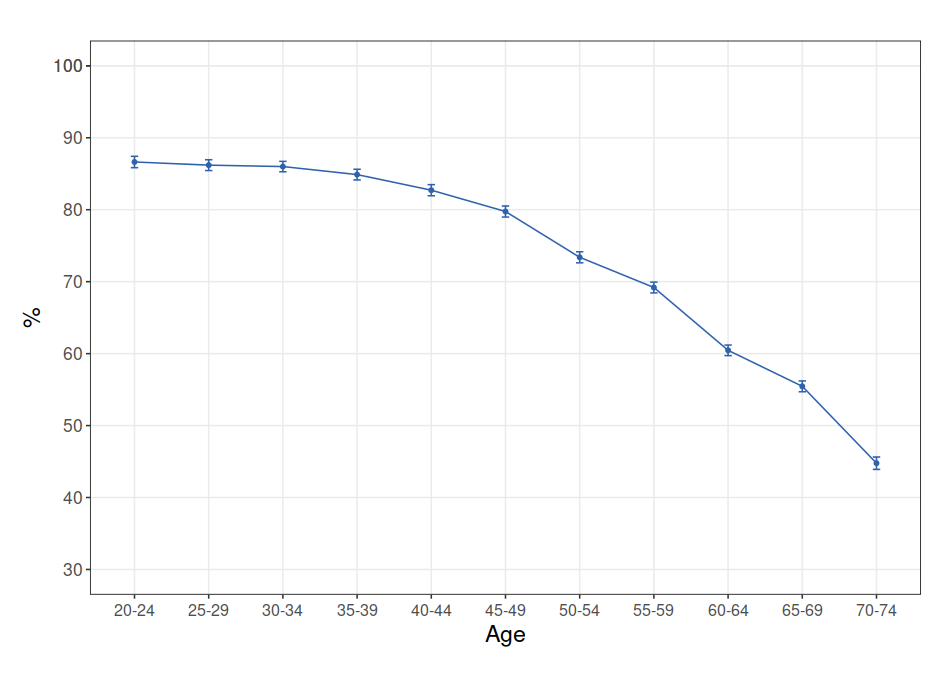


Supplementary figure 4. Prevalence of good work ability by five-year age groups.
